# Fentanyl Utilization During Extracorporeal Membrane Oxygenation and Risk of Methadone Treatment Among Pediatric ECMO Survivors

**DOI:** 10.1007/s00383-025-06267-5

**Published:** 2026-06-08

**Authors:** Rabab M. Barq, Shadassa Ourshalimian, Olivia A. Keane, Lara P. Nelson, Ashwini Lakshmanan, Henry C. Lee, Eugene Kim, Susan R. Hintz, Asavari Kamerkar, Lorraine I. Kelley-Quon

**Affiliations:** 1 Division of Pediatric Surgery, Children’s Hospital Los Angeles, 4650 Sunset Blvd, Mailstop #100, Los Angeles, CA 90027 USA; 2 Department of Anesthesiology and Critical Care Medicine, Children’s Hospital Los Angeles, Los Angeles, CA USA; 3 Division of Pain Medicine, Department of Anesthesiology Critical Care Medicine, Children’s Hospital Los Angeles, Los Angeles, CA USA; 4 Department of Pediatrics, Keck School of Medicine, University of Southern California, Los Angeles, CA USA; 5 Fetal and Neonatal Institute, Division of Neonatology, Department of Pediatrics, Keck School of Medicine, Children’s Hospital Los Angeles, University of Southern California, Los Angeles, CA USA; 6 Department of Health Systems Science, Bernard J. Tyson Kaiser Permanente School of Medicine, Pasadena, CA USA; 7 Division of Neonatology, University of California San Diego, La Jolla, CA USA; 8 Department of Pediatrics, Division of Neonatology, Stanford University School of Medicine, Palo Alto, CA USA; 9 Department of Population and Public Health Sciences, Keck School of Medicine, University of Southern California, Los Angeles, CA USA; 10 Department of Surgery, Keck School of Medicine, University of Southern California, Los Angeles, CA USA

**Keywords:** Extracorporeal membrane oxygenation (ECMO), Fentanyl, Methadone, Critical illness, Opioid dependence, Opioid tolerance, Opioid withdrawal

## Abstract

**Introduction:**

Single center studies suggest utilization of fentanyl during extracorporeal membrane oxygenation (ECMO) may be associated with a higher risk of opioid withdrawal. This study evaluated fentanyl use during ECMO and subsequent methadone or buprenorphine treatment among pediatric ECMO survivors in a large multi-center cohort.

**Methods:**

This retrospective study included children < 18y treated at 41 U.S. children’s hospitals in the Pediatric Health Information System between 2013 and 2023. Multivariable hierarchical regression was used to assess the relationship between fentanyl exposure on ECMO and likelihood of receiving methadone or buprenorphine after ECMO, adjusting for covariates including other opioid exposures.

**Results:**

Overall, 4,365 children were included (55.5% male; 47.9% neonatal). Median duration of ECMO was 5 (IQR: 3–8) days. Fentanyl exposure was categorized into quartiles:0–1 days, 2 days, 3–4 days, and ≥ 5 days. On multivariable regression, children in the 3–4 days quartile (OR 1.32; 95% CI: 1.05–1.65) and ≥ 5 days quartile (OR 2.21; 95% CI: 1.71–2.85) were more likely to receive methadone or buprenorphine after ECMO compared to those in the 0–1 days quartile. On bivariate comparison, children who received fentanyl during ECMO were found to have prolonged ventilator dependence, TPN use, and post-ECMO length of stay.

**Conclusion:**

Patients receiving fentanyl while on ECMO had an increased risk of receiving methadone or buprenorphine treatment post-ECMO, suggesting a higher risk of opioid withdrawal in patients receiving fentanyl while on ECMO. Our findings underscore a need for expanded opioid stewardship initiatives to utilize alternative pain management and minimize fentanyl prescribing for children undergoing ECMO.

## Introduction

Extracorporeal membrane oxygenation (ECMO) is an essential, life-saving intervention for critically ill pediatric patients with cardiac and/or respiratory failure; however, optimal management of sedation and analgesia during ECMO remains a significant clinical challenge. Fentanyl is frequently administered to critically ill children due to its quick onset of action and easily adjustable dosing. However, its pharmacokinetic profile is markedly altered by the ECMO circuit, which leads to significant sequestration into the circuit [1–4]. This sequestration within the ECMO circuit leads to unpredictable drug delivery, necessitating increasing doses to maintain adequate sedation, especially during initiation of ECMO and after circuit changes [1–3, 5–8]. Consequently, this increases the risk for oversedation, opioid dependence, and withdrawal, frequently necessitating treatment with weaning agents such as methadone or buprenorphine [9–12] .

Furthermore, fentanyl utilization during ECMO, extended use of any opioids, and the need for treatment of opioid dependence and withdrawal all contribute to prolonged hospital stays [7–9] and increased healthcare expenditures [13, 14]. Additionally, excess opioid exposure has been associated with a potential increased risk of neurodevelopmental impairment in infants [15–17]. Several investigations have attempted to evaluate the pharmacokinetics and clinical outcomes associated with fentanyl use during ECMO [1, 5, 7, 8]. Prior ex vivo work has demonstrated extensive fentanyl loss within ECMO circuits, with mean recovery as low as 3% compared to higher recoveries for midazolam (13%) and morphine (100%) [1, 5]. These findings suggest that fentanyl’s physicochemical properties may limit its effectiveness in ECMO-supported patients. Despite these efforts, limitations such as small sample sizes, focus on narrowly defined patient populations (e.g., neonates), and limited studies with contemporary cohorts have left key questions regarding the relationship between fentanyl exposure duration and post-ECMO opioid withdrawal largely unanswered [3, 7, 8]. Delirium has also been reported among pediatric patients supported with ECMO, although available evidence remains limited and inconsistent [18, 19].

Considering the pharmacokinetic challenges associated with fentanyl utilization for patients on ECMO, and the growing recognition of the potential neurocognitive and withdrawal sequelae of prolonged opioid exposure, the objective of this study was to evaluate the length of fentanyl exposure during ECMO and subsequent risk of methadone or buprenorphine treatment after decannulation. Findings of this study are intended to inform the development of standardized sedation and analgesic protocols, ultimately improving outcomes for children on ECMO.

## Methods

### Data Collection & Study Population

This multi-institutional retrospective cohort study utilized the Pediatric Health Information System (PHIS) database to identify patients less than 18-years-old who underwent ECMO cannulation between January 1, 2016 and December 31, 2023. The PHIS is an administrative database that aggregates encounter-level data from a network of not-for-profit, tertiary care pediatric hospitals across the United States. Data are de-identified upon submission and undergo rigorous quality assurance by both the Children’s Hospital Association (Lenexa, KS), and the participating institutions. Detailed information such as demographics, diagnostic and procedure codes, and resource utilization (e.g., pharmacy, imaging, laboratory data) is also collected by PHIS. This study was approved by the Institutional Review Board at Children’s Hospital Los Angeles, with a waiver of informed consent granted due to the use of de-identified data. This study also followed the Strengthening the Reporting of Observational Studies in Epidemiology (STROBE) reporting guidelines [20].

Patients were identified using Clinical Transaction Codes (CTC 521180 or 521181) and International Classification of Disease version 10 (ICD-10) codes for ECMO (5A15223, 5A1522F, 5A1522G, 5A15A2F, 5A15A2G, 5A1522H, 5A15A2H) [21–23]. We excluded patient encounters with ECMO durations of one day or less and those undergoing multiple ECMO courses. Children with exposure to methadone or buprenorphine preceding the ECMO course or a ICD-10 code for a diagnosis of opioid use disorder were excluded to ensure that any post-ECMO methadone or buprenorphine utilization was not for the treatment of pre-existing opioid dependence. Children who experienced in-hospital mortality during the index admission were excluded to facilitate evaluation of post-ECMO outcomes. Lastly, hospitals with known CTC data discrepancies were excluded. For example, two hospitals recorded ECMO charges only for repositioning or decannulation procedures, which prevented accurate assessment of ECMO duration, and another hospital was included only from 2020 onward following changes that separated ECMO charges from room charges. Additionally, data from certain years were omitted for one included hospital due to known inaccuracies prior to an update of their electronic medical record system.

Demographic variables analyzed included age, gender, race/ethnicity, and insurance status. Age at the time of ECMO cannulation was categorized into four groups: Neonates (< 28 days), infants (≥ 28 days to ≤ 1 year), pediatric (> 1 to ≤ 12 years) and adolescents (> 12 to < 18 years). Race was classified as Asian, Black, White, Unknown, or “Other” (the latter encompassing American Indian, Pacific Islander, and ‘Other’), while ethnicity was classified as Hispanic, non-Hispanic, or Unknown. Insurance status was also obtained from PHIS and was categorized as public, private or other. We reported sex, race/ethnicity, and insurance status given prior evidence that these demographic factors can influence prescribing practices among pediatric populations [24–31]. Weight data had a high degree of missingness in PHIS and were not analyzed.

As a proxy for medical illness severity, complex chronic conditions (CCCs) as defined by Feudtner et al. were reported [32]. Complex chronic conditions (CCCs) were defined using the Feudtner classification system, which identifies long-term pediatric conditions expected to persist for at least one year and require specialty or tertiary care. CCCs include cardiovascular disease, congenital or genetic disorders, gastrointestinal disorders, malignancies, metabolic disorders, neurologic or neuromuscular disorders, renal or urologic disorders, prematurity, and respiratory disease. Prematurity was defined by Feudtner et al. as birth occurring before 37 weeks of gestation.

## Exposures & Outcome Measures

Duration of fentanyl days during ECMO course was the primary exposure of interest. The duration of ECMO was measured from billing service dates, with first service date defined as day 1 (cannulation) and the last service date as the final day service date (decannulation). Any fentanyl administered on the same day as the billing service date for ECMO cannulation or decannulation was considered given during the ECMO episode. Duration of fentanyl days was categorized into quartiles: 1st quartile (0–1 days), 2nd quartile (2 days), 3rd quartile (3–4 days), and 4th quartile (≥ 5 days).

The primary outcome of interest was the receipt of methadone or buprenorphine after decannulation from ECMO (but during the same hospitalization). Receipt of methadone or buprenorphine was assumed to be a proxy measurement for opioid dependence or iatrogenic withdrawal requiring weaning agents. Treatment duration of methadone or buprenorphine was not analyzed, as prior studies have shown that inpatient outcomes such as TPN use and mechanical ventilation are impacted by the presence of methadone exposure rather than its duration [9]. Secondary outcomes included post-ECMO decannulation days, requiring mechanical ventilation, and days of total parenteral nutrition (TPN) use, and length of hospital stay after the ECMO course were also assessed. Lastly, factors associated with fentanyl exposure during ECMO were also assessed.

Co-variates included patient sex, age, race and ethnicity, insurance status, hospital region, CCCs, pre-ECMO opioid exposure, and other opioid exposure during ECMO (morphine, hydromorphone, and oxycodone). Weight was not collected due to the high degree of missingness in PHIS. Pre-ECMO opioid exposure was defined as any opioid exposure before or on the day of ECMO cannulation. Other opioid exposure during ECMO was similarly defined as fentanyl exposure during ECMO and included morphine, hydromorphone, and oxycodone.

### Statistical Analysis

Continuous variables were described using medians and interquartile ranges (IQR) and were analyzed using Wilcoxon-Mann-Whitney tests. Categorical variables were reported as counts and percentages and were analyzed using Chi-squared or Fischer’s exact tests as appropriate. Bivariate analyses were performed to evaluate crude differences in patient and hospital characteristics between patients that were not exposed and those that were exposed to fentanyl during ECMO. Post-ECMO days requiring mechanical ventilation, days of R use, and length of stay were calculated for patients who did and did not receive fentanyl during their ECMO course. Days were then compared between groups using Kruskal-Wallis tests.

Multiple imputation using chained equations was utilized to address missing data for race and ethnicity. This was performed under the assumption that missing data were missing at random. Patient and hospital characteristics including hospital region, sex, age, insurance status, and CCCs were used for imputation. Multivariable hierarchical logistic regression modeling was used to assess the relationship between fentanyl exposure quartiles and likelihood of receiving methadone or buprenorphine after ECMO, using a binomial distribution and logit link. Multivariable hierarchical logistic regression modeling was also used to assess other factors associated with methadone or buprenorphine treatment after ECMO. Covariates were selected a priori and included patient sex, age, race and ethnicity, insurance status, hospital region, CCCs, pre-ECMO opioid exposure, and other opioid exposure during ECMO (morphine, hydromorphone, and oxycodone). Model convergence and fit were assessed using standard diagnostic measures Akaike Information Criterion (AIC) and Bayesian Information Criterion (BIC). To account for hospital-level clustering, patients were nested within hospitals, with hospitals considered a random effect, and patient factors were considered as fixed effects to account for unmeasured institutional differences [33]. Odds ratios (ORs) and 95% Wald confidence intervals (CI) were reported. A p-value of < 0.05 was considered statistically significant. All analyses were performed using SAS version 9.4 (SAS Institute, Cary, NC) and Stata version 15.

## Results

**Fig. 1:**
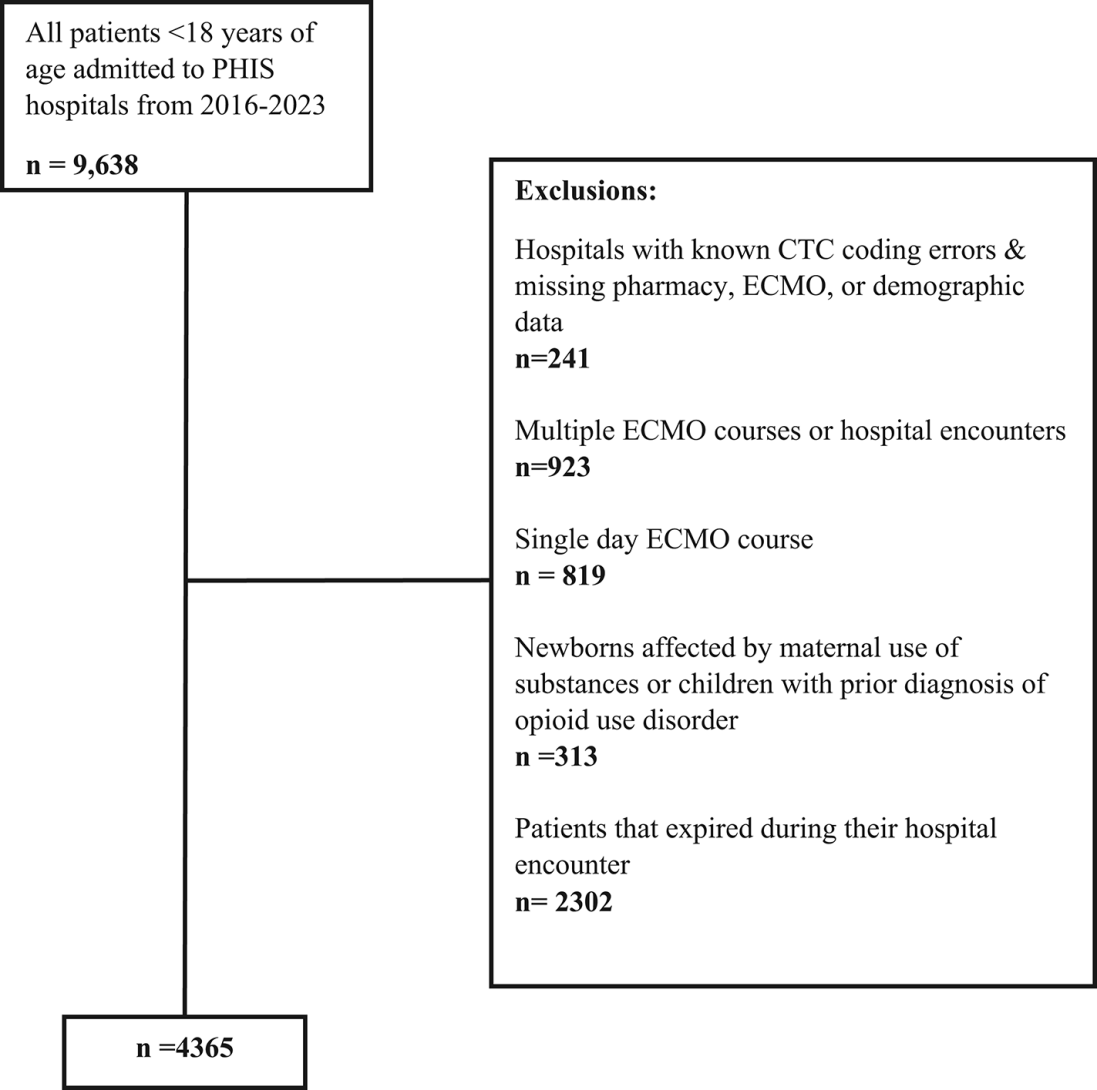
Study Flow Diagram

A total of 4,365 children from 41 children’s hospitals were included in this study (Fig. 1). Of these, 55.5% were male, 54.7% identified as White, and 76.9% were non-Hispanic (Table 1). Neonates comprised 47.9% of the cohort, followed by pediatric patients (22.9%), infants (17.4%) and adolescents (11.8%). The majority of our cohort had public insurance (55.0%). The most common CCC was cardiovascular disease, occurring in 69.2% of patients. Additionally, 43.2% of the cohort had three or more CCCs. Most patients were exposed to ≤ 7 days of opioids preceding their ECMO course (88.7%). The median duration of ECMO was 5 days (IQR: 3–7). Overall, most patients were exposed to fentanyl during their ECMO course (92.5%), and a smaller proportion of patients received methadone or buprenorphine after their ECMO course (38.2%). Of the patients that received post-ECMO methadone or buprenorphine treatment, 94.4% had received fentanyl during their ECMO course. ECMO duration itself was clinically similar between patients who did and did not receive fentanyl during their ECMO course (median: 5 (IQR 3–8) days vs. 5 (IQR 3–7).

**Table 1:** Clinical and demographic factors stratified by Fentanyl exposure during ECMO

	Cohort	No Fentanyl	Any Fentanyl	*p*-value
	*N* = 4365 (%)	*N* = 329 (%)	*N* = 4036 (%)	
**Age**				< 0.009
Neonates (0-<28d)	2092 (47.9)	129 (39.2)	1963 (48.6)	
Infants (≥ 28d to ≤ 1 year)	759 (17.4)	66 (20.1)	693 (17.2)	
Pediatric (> 1 to ≤ 12yrs)	1000 (22.9)	93 (28.3)	907 (22.5)	
Adolescent (> 12 to < 18)	514 (11.8)	41 (12.5)	473 (11.7)	
**Sex**				0.334
Female	1942 (44.5)	138 (41.9)	1804 (44.7)	
Male	2423 (55.5)	191 (58.1)	2232 (55.3)	
**Race**				0.327
Black	850 (19.5)	60 (18.2)	790 (19.6)	
Asian	126 (2.9)	10 (3)	116 (2.9)	
White	2386 (54.7)	192 (58.4)	2194 (54.4)	
Other	740 (17)	45 (13.7)	695 (17.2)	
Unknown	263 (6)	22 (6.7)	241 (6)	
**Ethnicity**				0.752
Hispanic	704 (16.1)	51 (15.5)	653 (16.2)	
Not Hispanic	3358 (76.9)	258 (78.4)	3100 (76.8)	
Unknown	303 (6.9)	20 (6.1)	283 (7)	
**Insurance**				0.312
Private	1835 (42)	126 (38.3)	1709 (42.3)	
Public	2400 (55)	191 (58.1)	2209 (54.7)	
Other	130 (3)	12 (3.6)	118 (2.9)	
**Other opioids**				
Hydromorphone	1041 (23.8)	110 (33.4)	931 (23.1)	< 0.001
Morphine	3027 (69.3)	237 (72)	2790 (69.1)	0.271
Oxycodone	61 (1.4)	4 (1.2)	57 (1.4)	0.999
**Pre-ECMO opioid exposure**				0.003
≤ 7d	3871 (88.7)	279 (84.8)	3592 (89)	
> 7 or ≤ 14d	314 (7.2)	39 (11.9)	275 (6.8)	
> 14d	180 (4.1)	11 (3.3)	169 (4.2)	
**CCCs**				
Cardiovascular	3022 (69.2)	187 (56.8)	2835 (70.2)	< 0.001
Congenital or Genetic	938 (21.5)	56 (17)	882 (21.9)	0.040
Gastrointestinal	1321 (30.3)	98 (29.8)	1223 (30.3)	0.845
Malignancy	123 (2.8)	16 (4.9)	107 (2.7)	0.020
Metabolic	853 (19.5)	68 (20.7)	785 (19.4)	0.592
Neurologic/Neuromuscular	836 (19.2)	58 (17.6)	778 (19.3)	0.465
Premature	1686 (38.6)	137 (41.6)	1549 (38.4)	0.243
Renal and Urologic	885 (20.3)	61 (18.5)	824 (20.4)	0.416
Respiratory	873 (20)	59 (17.9)	814 (20.2)	0.330
**CCC count**				0.076
0	252 (5.8)	28 (8.5)	224 (5.6)	
1	1034 (23.7)	86 (26.1)	948 (23.5)	
2	1193	83	1110	
3	1886	132	1754	

*CCC * complex chronic conditions, *ECMO* extracorporeal membrane oxygenation

We divided fentanyl exposure into quartiles by ECMO-days: Q1 0–1 days, Q2 2 days, Q3 3–4 days, Q4 ≥ 5 days. On unadjusted analysis of fentanyl quartiles using the 1st and lowest quartile as the reference, the odds ratios (OR) for receiving post-ECMO methadone or buprenorphine were 0.97 (95% CI, 0.79–1.20; *p* = 0.805) for the 2nd quartile, 1.25 (95% CI, 1.01–1.55; *p* = 0.038) for the 3rd quartile, and 2.08 (95% CI, 1.65–2.64; *p* < 0.001) for the 4th quartile. After adjustment for other opioid exposures during ECMO, pre-ECMO opioid exposure, age, race, ethnicity, insurance, region, and CCCs, the 3rd and 4th quartiles remained significantly associated with the post-ECMO methadone and buprenorphine—adjusted OR 1.32 (95% CI, 1.05–1.65; *p* = 0.017) and adjusted OR 2.21 (95% CI, 1.71–2.85; *p* < 0.001), respectively (Table 2). The 2nd quartile did not differ significantly from the reference (*p* = 0.920).

**Table 2:** Multivariable hierarchical logistic regression identifying factors associated with post-ECMO methadone or buprenorphine treatment

	OR (95% CI)	*p*-value
**Fentanyl quartiles**		
2 days	1.01 (0.81–1.26)	0.92
3–4 days	1.32 (1.05–1.65)	0.017
≥ 5 days	2.21 (1.71–2.85)	< 0.001
0–1 days	ref	ref
**Other opioids**		
Hydromorphone	1.83 (1.48–2.26)	< 0.001
Morphine	1.18 (0.98–1.43)	0.081
Oxycodone	0.33 (0.15–0.73)	0.006
**Pre-ECMO opioid days**		
> 14d	1.68 (1.16–2.43)	0.007
> 7 or ≤ 14d	1.87 (1.41–2.50)	< 0.001
≤ 7d	ref	ref
**Age**		
Neonates (< 28d)	1.34 (0.97–1.83)	0.073
Infants (≥ 28d to ≤ 1 year)	4.08 (3.04–5.48)	< 0.001
Pediatric (> 1 to ≤ 12yrs)	2.54 (1.94–3.33)	< 0.001
Adolescent (> 12 to < 18)	ref	ref
**Sex**		
Female	0.98 (0.84–1.13)	0.747
Male	ref	ref
**Race**		
AA/Black	0.72 (0.58–0.89)	0.003
Asian	0.92 (0.59–1.49)	0.789
Other	0.87 (0.70–1.12)	0.295
White	ref	ref
**Ethnicity**		
Hispanic	1.01 (0.80–1.29)	0.905
Not Hispanic	ref	ref
**Insurance**		
Private	0.88 (0.75–1.04)	0.131
Other	1.11 (0.72–1.70)	0.633
Public	ref	ref
**Region**		
Midwest	2.01 (0.67–6.07)	0.215
Northeast	0.94 (0.24–3.71)	0.934
South	3.10 (1.08–8.93)	0.036
West	ref	ref
**CCCs**		
Cardiovascular	1.17 (0.99–1.39)	0.068
Congenital or Genetic	0.84 (0.68–1.03)	0.088
Gastrointestinal	1.29 (1.09–1.54)	0.004
Malignancy	0.84 (0.54–1.31)	0.448
Metabolic	1.32 (1.08–1.61)	0.007
Neurologic/Neuromuscular	1.04 (0.86–1.25)	0.722
Premature	1.04 (0.84–1.30)	0.707
Renal and Urologic	1.27 (1.05–1.53)	0.012
Respiratory	1.40 (1.14–1.73)	0.002

The multivariable analysis identified several factors associated with post-ECMO methadone or buprenorphine treatment, holding other covariates constant (Table 2). Hydromorphone exposure during ECMO was significantly associated with increased odds of methadone or buprenorphine treatment post-ECMO (OR 1.83; 95% CI, 1.48–2.26; *p* < 0.001), while any oxycodone exposure during ECMO was associated with reduced odds of post-ECMO methadone/buprenorphine (OR 0.33; 95% CI, 0.15–0.73; *p* = 0.006). Compared with those with ≤ 7 days of pre-ECMO opioid use, patients with more than 14-days of pre-ECMO opioid exposure had an increased likelihood of post-ECMO methadone or buprenorphine treatment (OR 1.68; 95% CI, 1.16–2.43; *p* = 0.007), as did those with > 7 and ≤ 14 days (OR 1.87; 95% CI, 1.41–2.5; *p* < 0.001). Compared to adolescents, pediatric patients (OR 2.5; 95% CI: 1.94–3.33; *p* < 0.001) and infants (OR 4.08; 95% CI: 3.04–5.48; *p* < 0.001) had increased risk of post-ECMO methadone or buprenorphine treatment. Regional differences were noted, with patients in the South showing higher odds (OR 3.10; 95% CI: 1.08–8.93; *p* = 0.036) relative to those in the West. Gastrointestinal (OR 1.29; 95% CI: 1.09–1.54; *p* < 0.001), metabolic (OR 1.32; 95% CI: 1.08–1.61; *p* = 0.007), renal/urologic (OR 1.27; 95% CI: 1.05–1.53; *p* = 0.012), and respiratory (OR 1.4; 95% CI: 1.14–1.73; *p* = 0.002) conditions were significantly associated with increased likelihood of post-ECMO methadone or buprenorphine treatment.

On bivariate comparison of secondary health outcomes of interest, patients who received fentanyl during ECMO were found to have prolonged ventilator dependence, TPN use, and post-ECMO length of stay (Table 3). Patients who received fentanyl during their ECMO course required mechanical ventilation for a median of 8 days (IQR, 4–19) versus 7 days (IQR, 4–16) (*p* = 0.025), TPN for a median of 18 days (IQR, 9–14) versus 14 days (IQR, 6–29) (*p* < 0.001), and had a median post-ECMO length of stay of 34 days (IQR, 18–61) versus 27 days (IQR, 16–49) (*p* < 0.001).

**Table 3:** Post-ECMO outcomes associated with Fentanyl exposure during ECMO course

	Median (IQR)	Received Fentanyl while on ECMO		*p*-value
		Yes	No	
**Ventilator use, days**	8 (4–19)	8 (4–19)	7 (4–16)	0.050
**TPN use,days**	18 (9–14)	18 (9–42)	14 (6–29)	< 0.001
**Post-ECMO length of stay, days**	34 (18–61)	34 (18–64)	27 (16–49)	< 0.001
**Length of ECMO course, days**	5 (1-190)	5 (3–8)	5 (3–7)	0.869

## Discussion

In this multicenter cohort study across U.S. children’s hospitals, we identified a significant association between receipt of fentanyl during ECMO and risk of later methadone or buprenorphine treatment in pediatric ECMO survivors. Additionally, any hydromorphone exposure during ECMO was associated with a higher likelihood of requiring post-ECMO methadone or buprenorphine treatment, while oxycodone exposure (although infrequent) was associated with a reduced likelihood of treatment. Younger children, particularly infants, had a higher likelihood of requiring post-ECMO methadone or buprenorphine compared to adolescents. Finally, children who were received fentanyl during their ECMO course were more likely to experience prolonged ventilator dependence, TPN use, and hospital stays, suggesting broader implications for resource utilization and patient outcomes.Our findings provide compelling evidence to support minimizing fentanyl utilization for patients on ECMO.

Our study demonstrated a significant association between fentanyl exposure duration and increased likelihood of post-ECMO methadone or buprenorphine treatment. Due to its lipophilic and protein-binding properties, fentanyl is extensively sequestered within ECMO circuits, particularly in membrane oxygenators and roller-head pumps [1–6, 34–37]. This process reduces the drug’s availability in circulation, often requiring higher starting doses and frequent titrations to maintain adequate analgesia and sedation [1, 3]. Our findings align with previous smaller, single-center studies indicating that fentanyl exposure leads to increased risk of opioid tolerance and withdrawal in critically ill infants and children [7, 8]. By confirming and expanding on these findings in a larger multicenter cohort, our study provides further evidence to support the minimization of fentanyl use in ECMO patients. Future research should prioritize developing and implementing standardized guidelines for fentanyl-sparing sedation and analgesic strategies for patients on ECMO while monitoring for improved patient outcomes. Our analysis specifically examined fentanyl exposure during ECMO and the subsequent need for methadone or buprenorphine treatment, rather than evaluating other sedative or analgesic medications. Sedation and analgesic regimens during ECMO are inherently complex and may contribute to variability in both withdrawal and delirium risk. Although our analysis focused on fentanyl exposure, we did not examine concurrent sedative or analgesic medications, such as hydromorphone, morphine, or dexmedetomidine, nor the incidence of withdrawal or delirium related to these medications, which may influence these outcomes. Recent studies have reported differing opioid and sedative dosing patterns by medication type and time of day in ECMO patients [38, 39]. Future work should evaluate how medication selection, dosing variability, and institutional sedation practices together affect withdrawal and sedation-related outcomes. Additionally, while pre-ECMO ventilation and sedation data are available within PHIS, these variables were not collected or included in our analysis because our study focused on opioid exposure during ECMO and subsequent withdrawal treatment rather than pre-cannulation management.

The observed association between hydromorphone exposure and an increased risk methadone or buprenorphine treatment, contrasted with oxycodone’s protective effect, suggests that opioid selection may play a critical role in post-ECMO withdrawal outcomes. Notably, morphine did not have a significant impact on risk of post-ECMO methadone or buprenorphine treatment. Hydromorphone, like fentanyl, exhibits lipophilic and protein-binding properties, resulting in sequestration within the ECMO circuit [3, 37]. As a result, higher doses may be required to achieve therapeutic effects, increasing the risk of opioid dependence and withdrawal. However, despite these challenges, because hydromorphone undergoes less sequestration than fentanyl within the ECMO circuit, it remains a viable alternative for pain management [3]. A retrospective study assessing hydromorphone versus fentanyl in ECMO patients found that hydromorphone use was linked to reduced opioid requirements post-ECMO without significant differences in sedation needs or efficacy of pain control [37]. Currently, there is a paucity of literature examining the use of oxycodone, or any oral medications, for ECMO patients. Generally, orally administered medications are avoided in critically ill patients due to evidence of erratic gastrointestinal absorption [40–43]. However, our findings could suggest that oxycodone may offer advantages over fentanyl and hydromorphone, potentially allowing for smoother weaning and lower withdrawal risk. Alternatively, these findings may only reflect patients who are weaning from ECMO and thus able to tolerate enteral medications and may not represent a true protective effect of oxycodone itself. Additionally, morphine demonstrates more stable pharmacokinetics than fentanyl, with considerably less sequestration and a more reliable clearance process [3, 36]. Studies indicate that morphine effectively manages pain while being associated with a lower incidence of withdrawal symptoms after ECMO when compared to fentanyl, with some researchers advocating for its use as the preferred opioid in neonatal ECMO [7, 44] However, morphine is metabolized in the liver into active glucuronide derivatives, including morphine-6-glucuronide, which can accumulate in patients with renal dysfunction, potentially leading to prolonged sedation and respiratory depression, thereby limiting its suitability for those with severe hepatic or renal impairment [36, 44, 45]. Currently, there is limited robust literature evaluating the safety and efficacy of alternative sedative and analgesic agents in pediatric ECMO patients [3]. However, medications such as dexmedetomidine, ketamine, and acetaminophen have all been proposed as potential alternatives to allow for minimizing excess opioid and benzodiazepine use, which carry risks of altered pharmacokinetics and serious adverse effects [3]. Data are still emerging on the short- and long-term effects of all these sedating medications on the developing brain and risk for cognitive dysfunction. Ultimately, further research is needed to ascertain the safety and efficacy of individual analgesic agents for patients on ECMO.

Our study also found that infants and pediatric patients had significantly higher odds of requiring post-ECMO methadone or buprenorphine treatment as compared to adolescents, suggesting an age-dependent vulnerability to opioid withdrawal [46]. This finding is consistent with prior research indicating that younger critically ill patients in pediatric intensive care units, particularly infants under six months of age, are more likely to experience opioid withdrawal [47]. Immature hepatic and renal enzymes prolong drug clearance in infants and neonates, leading to extended receptor activation and an increased likelihood of developing tolerance and dependence [47–49]. Clinicians should consider these age-specific pharmacokinetic differences when prescribing opioids to young pediatric patients to mitigate the risk of withdrawal and dependence.

Lastly, children who received fentanyl during ECMO experienced significantly longer mechanical ventilation duration, extended TPN use, and prolonged post-ECMO hospital stays. These findings align with previous studies suggesting that excessive opioid exposure can cause a depressed respiratory drive, leading to difficulty weaning from mechanical ventilation [50, 51], gut dysmotility requiring prolonged TPN use [52], and prolonged length of stay to treat the aforementioned complications [51, 53, 54]. However, these associations should be interpreted as correlational rather than causal, as prolonged ventilation and length of stay may also reflect greater underlying illness severity or complexity among patients requiring longer or higher-dose opioid exposure. Residual confounding by indication or disease severity cannot be excluded. Specifically, fentanyl utilization for patients on ECMO carries a particularly higher risk of opioid dependence and prolonged length of stay when compared to other opioids [7, 49]. Given the implications for patient outcomes and increased healthcare utilization [9], future research should explore multimodal pain management strategies that optimize sedation while minimizing the risks of prolonged opioid exposure.

This study has several limitations. First, because PHIS is an administrative database that primarily includes children’s hospitals, our findings may not fully represent fentanyl prescribing patterns in other healthcare settings, such as adult hospitals that care for children. This selection bias may limit transportability of our findings to other target populations. However, PHIS is one of the few administrative databases that include detailed pharmacy data, allowing for a broad evaluation of opioid utilization in high-acuity centers that frequently manage ECMO patients. Additionally, our analysis was restricted to inpatient survivors, as children who died during hospitalization were excluded from the cohort. Therefore, “atient outcomes in this study reflect inpatient morbidity measures—such as duration of mechanical ventilation, TPN use, and hospital length of stay—rather than mortality or post–ICU syndrome. Second, ECMO duration was determined using CTC billing codes, which are subject to variability in institutional billing practices. This could lead to imprecise estimations of ECMO cannulation and decannulation dates. To minimize misclassification, we excluded 232 patients from hospitals where discrepancies in CTC coding were identified in collaboration with PHIS data analysts. Our analysis was also limited to inpatient outcomes available within PHIS. In this context, “patient outcomes” reflect markers of hospital morbidity—such as duration of mechanical ventilation, TPN use, and hospital length of stay—rather than mortality or post–ICU syndrome, as we excluded patients who did not surive until discharge. Additionally, PHIS does not distinguish between venoarterial (VA) and venovenous (VV) ECMO, which may introduce residual confounding because ECMO mode is determined by indication and illness severity. However, pharmacokinetic studies suggest that drug sequestration within ECMO circuits is primarily influenced by circuit surface area and drug characteristics—such as lipophilicity and protein binding—rather than by VA or VV configuration [1, 5] .Next, PHIS lacks detailed fentanyl dosing information, preventing differentiation between a single dose and a continuous infusion administered within the same day. This limits our ability to precisely characterize fentanyl exposure among patients and across institutions. PHIS does not include device-level ECMO details such as oxygenator or pump type and day-by-day circuit characteristics. Although PHIS includes pre-ECMO ventilation and sedation data, these variables were not collected for this study given our focus on opioid exposure during ECMO and subsequent withdrawal treatment. These omissions limit the ability to assess how patient size, circuit configuration, or pre-ECMO sedation practices may have influenced drug kinetics or exposure duration. Additionally, PHIS does not capture bedside sedation scores, comprehensive concurrent sedative or adjunct medication details, or physiologic and laboratory parameters such as serum creatinine, urea, albumin, or liver function tests, limiting our ability to account for sedation depth and illness severity. Despite this, administrative billing data can provide a meaningful epidemiologic overview of fentanyl exposure in routine ECMO care. Additionally, because PHIS captures only daily medication administration without precise timestamps, there is a possibility that fentanyl prescribed before ECMO cannulation or after decannulation (if occurring on the same day) was misclassified as ECMO-related exposure. However, this type of misclassification is likely non-differential across institutions. As a result, while it may slightly dilute our observed associations, it is unlikely to introduce significant systematic bias and substantially alter estimates between fentanyl and post-ECMO weaning agents. Lastly, methadone and buprenorphine treatment were used as proxies for opioid withdrawal and dependence, as they are commonly employed for these conditions [10–12]. However, institutional protocols often dictate methadone or buprenorphine initiation based on cumulative opioid exposure rather than clinical withdrawal symptoms alone [10]. While this proxy is imperfect, the use of a robust multivariable regression model with adjustment for key confounders allows us to detect meaningful variations in our outcome about the association between fentanyl exposure during ECMO and the likelihood of subsequent methadone or buprenorphine treatment.

## Conclusion

This multicenter study provides evidence for the association between fentanyl exposure during ECMO and post-ECMO methadone or buprenorphine treatment, suggesting an increased risk of opioid dependence and withdrawal. This association remained significant even after adjusting for pre-ECMO opioid exposure, the use of other opioid analgesics, and key demographic and clinical factors. Our findings further highlight the increased vulnerability of neonates and infants to potential opioid withdrawal, emphasizing the need for age-specific opioid stewardship efforts. Beyond withdrawal risk, fentanyl exposure was also associated with prolonged mechanical ventilation, extended TPN use, and longer hospital stays, reinforcing concerns about its impact on patient outcomes and healthcare resource utilization. Given the accumulating evidence on the risks of fentanyl in ECMO patients, efforts should focus on optimizing analgesic and sedation protocols to minimize fentanyl use while ensuring effective pain management and minimizing risk. Future research should explore alternative fentanyl-sparing strategies and assess the safety and efficacy of oral analgesics, such as oxycodone, in ECMO patients.

## Data Availability

No datasets were generated or analysed during the current study.
